# Personals

**Published:** 1912-11

**Authors:** 


### PERSONALS.

Dr. Charles W. Hennington of Rochester, Associate Editor, is compiling biographic data about Dr. E. G. Munn of Rochester, the first ophthalomologist in western New York. He lived from 1804 till Dec., 1847. We do not find an obituary notice in our file of the Journal. Any one having information about Dr. Munn will please communicate with Dr. Hennington.

Dr. A. E. Bartoo, University of Buffalo, 1888, long a practitioner of Nebraska, has recently returned from a year's absence in Brazil and has opened an office at 353 Glenwood Ave., Buffalo.

Dr. Arthur L. Hinman of Clyde has been appointed from the state civil service list as Assistant Surgeon to the State Soldiers' Home, succeeding Dr. Edward Tierney.

Dr. A. J. Martin of North Tonawanda will return about Nov. 1, from a six weeks' trip to the Pacific coast.

Dr. Robert P. Bush of Horseheads has again been nominated by the Democrats for member of the assembly.

Dr. E. T. Bush of Horseheads has gone to New York for four months' post graduate study.

Dr. F. St. J. Hoffman of Buffalo, has recently returned from Panama, South America and Jamaica.

---

Dr. Horace Lo Grasso, a former Buffalonian, leaves his service at Raybrook to become Resident Physician at the J. N. Adam Hospital at Perrysburg.

---

Dr. Edward C. Perry recently suffered considerable loss from the burning of his barn along with others. The fires are believed to have been of incendiary origin.

---

Dr. J. D. Zwetsch of Gowanda has recently returned from a trip to Chicago.

---

Dr. Max Keiser and Dr. Wm. M. Mehl of Buffalo have recently returned from Europe.

---

Dr. Joseph S. Lewis of Buffalo, spent a couple of weeks at Point Abino during October, wild ducks appearing in abundance the morning of his departure.

---

An epidemic of typhoid, probably water borne, is in progress in Troy Pa., and neighboring villages. Troy, with a population of 1500, had 172 cases up to Oct. 26. We have written for a special report but have had no response in time for the present issue of the JOURNAL.

